# Emerging role of mitoNEET as mitochondrial sensor of hypoxia

**DOI:** 10.1007/s00775-026-02139-y

**Published:** 2026-03-09

**Authors:** Nghi Thao Hoang, Kourosh Honarmand Ebrahimi

**Affiliations:** 1 Institute of Pharmaceutical Science, King’s College London, London, UK; 2 Department of Pharmacy, Da Nang University of Medical Technology and Pharmacy, Da Nang, Vietnam

**Keywords:** mitoNEET, Iron-sulfur cluster, Hypoxia, Mitochondria, Nitric oxide, Hydrogen sulfide

## Abstract

Mitochondria are organelles whose function and dynamics, that is fission, fusion, transport and selective removal (mitophagy), are fundamental for eukaryotic cells. Their dysfunction is associated with age-related neurodegeneration and the aging immune system. MitoNEET, an iron-sulfur (Fe-S) cluster-containing protein localised at the outer membrane of mitochondria, has emerged as a key regulator of mitochondrial respiration and linked to fission, fusion, and mitophagy. In this perspective article, we discuss recent emerging evidence describing the mitoNEET [2Fe-2S] cluster role as a sensor of the cellular level of O_2_. These findings provide a mechanistic explanation of how mitochondria sense hypoxia, which affects their bioelectrochemistry and dynamics. We hope our data and analysis stimulate future research to fully understand the chemistry and biology of this intriguing Fe-S protein. These studies are expected to reveal new mechanistic insight into mitochondrial function and dynamics and help develop new therapeutics for inflammatory and age-related diseases.

**Graphical Abstract:**

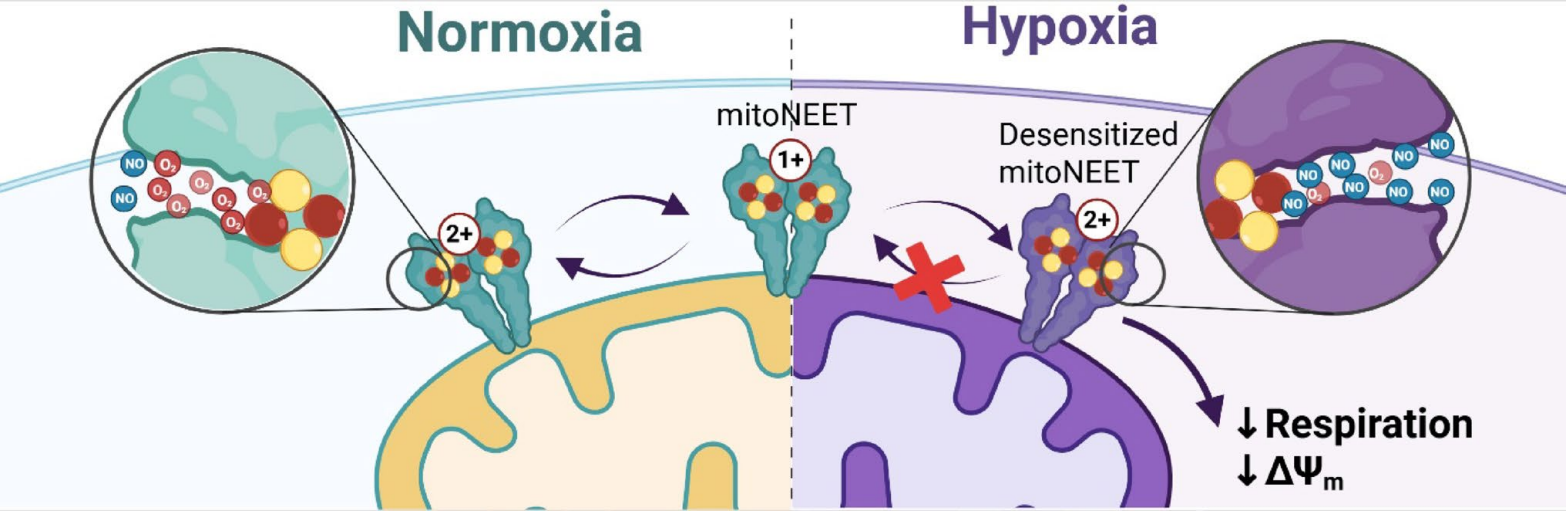

## Iron-sulfur protein in the immune system

**Fig. 1:**
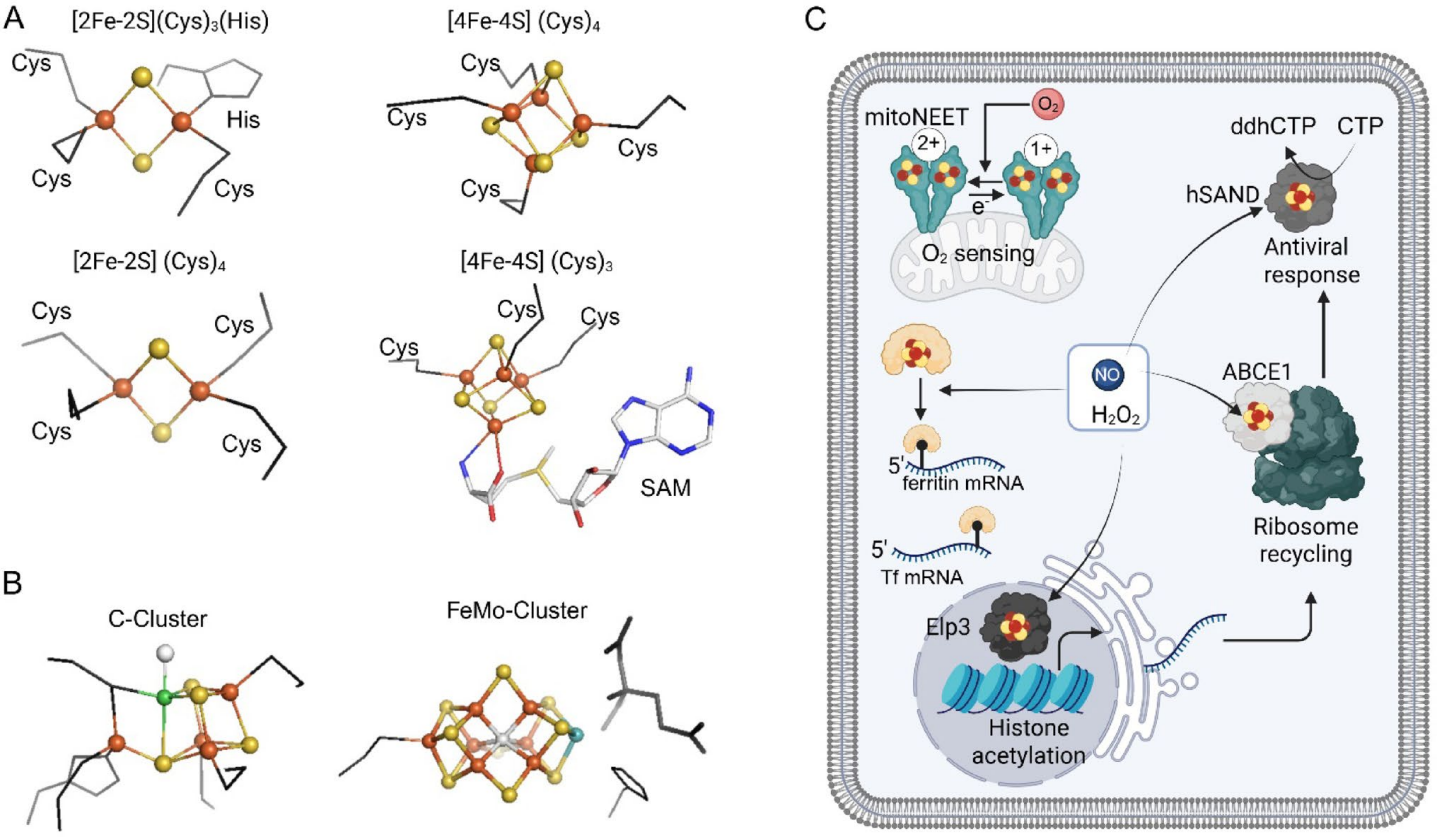
Iron-sulfur (Fe-S) clusters are diverse biological cofactors with emerging roles in the innate immune system. (**A**) Some of the most common Fe-S clusters are found in many cellular proteins, including those involved in the innate immune response. (**B**) Two types of complex Fe-S clusters, the C-cluster of carbon monoxide dehydrogenase and the FeMo-cluster of nitrogenase. (C) The [4Fe-4S] and [2Fe-2S] clusters found in proteins play a part in different steps of the innate immune system. Elp3 catalyzes the acetylation of histones to stimulate transcription of IFN mRNA. ABCE1 is involved in the ribosome recycling and translation of different proteins, including interferon-stimulated genes (ISGs). The ISG protein hSAND is a key antiviral response enzyme, and its [4Fe-4S] cluster is involved in the transformation of nucleoside triphosphates, such as CTP, to their antiviral nucleoside triphosphate analogue ddhNTP, such as ddhCTP. The [4Fe-4S] cluster in all these proteins and in responsive protein 1 (IRP1) acts as a biological fuse and is degraded by NO and H_2_O_2_. In contrast to the biological function of the Fe-S cluster, which leads to its degradation, our recent findings suggest that the mitoNEET [2Fe-2S] cluster is involved in O 2 sensing and is not degraded

Bioinorganic cofactors made of iron and sulfur, iron-sulfur (Fe-S) clusters, are among the most ancient and versatile cofactors in biology, essential for the emergence and maintenance of life on Earth [1]. They are found either as simple iron-sulfur complexes (Fig. 1A) or as more complex structures, such as the FeMo-cluster in nitrogenases (Fig. 1B) [2]. They vary in stoichiometry of iron and sulfur [1, 3]. In target proteins, these clusters can have different functions: electron transfer, e.g., in the respiratory complexes or hydrogenases [4–6], enzymatic catalysis, e.g., in radical S-adenosylmethionine (SAM) enzymes such as human radical-SAM dependent nucleotide dehydratase (hSAND) also known as RSAD2 or viperin [1, 7–10], structural stabilisation, e.g., in ABCE1 protein [11], sulfur donation, e.g., in some radical-SAM enzymes [12, 13], and sensing of cellular redox or reactive species, e.g., as a biological fuse, where the FeS cluster is degraded, in multiple Fe-S enzymes involved in the innate immune system [1]. Consequently, Fe-S clusters are essential not only for energy metabolism, respiration, and DNA repair, but also for regulatory networks that couple metabolic and environmental cues to cellular “decision-making”. The biogenesis and maintenance of Fe-S clusters are tightly regulated through complex Fe-S biogenesis and assembly pathways evolved early in evolution [14]. Proper cluster assembly ensures functional maturation of Fe-S proteins, enabling them to perform their biological roles. Conversely, oxidative stress-induced cluster damage can inactivate Fe-S enzymes and destabilise Fe-S protein complexes [15]. Therefore, Fe-S clusters are well-suited to act as molecular sensors, transducing redox, oxygen, or iron-availability signals into various cellular responses, such as the transcription or translation of specific genes and the modulation of metabolism.

Within the innate immune system, Fe-S proteins are involved in multiple steps, from pathogen recognition to transcription/translation of proinflammatory signals, activation of effector enzymes (Fig. 1 C) [1]. For instance, the Fe-S enzyme Elp3 (the catalytic subunit of the elongator complex) is involved in the acetylation of histones H3 and H4 [16], which could prime the innate immune system by recruiting RNA Pol II and promoting the transcription of proinflammatory cytokines such as type I interferons (IFNs) [1]. The resulting mRNAs are translated by the ribosome, whose function requires Fe-S protein ABCE1 [17, 18]. The Fe-S cluster in ABCE1 provides structural and functional stability. Finally, interferon-stimulated gene (ISG) protein SAM-dependent nucleotide dehydratase (SAND) (also known as RSAD2 or viperin in humans) [8, 10] is an Fe-S enzyme member of the radical-SAM superfamily [8]. The Fe-S cluster in human SAND (hSAND) is fundamental for the catalytic function of the enzyme, transforming cytosine triphosphate to its nucleotide analogue 3′-deoxy-3′,4′-didehydro cytidine triphosphate (ddhCTP) [19]. In addition to their roles in catalysis and structural stability, the Fe-S clusters in several proteins act as sensors of reactive oxygen or nitrogen species. Examples of these clusters include the [4Fe-4S] in IRP1, and the [2Fe-2S] cluster in FBXL5 [1]. The [4Fe-4S] cluster in IRP1 dictates the ability of the protein to recognise and bind to specific structural elements, named iron-responsive elements (IREs), at the 3´ or 5´ end of mRNA (Fig. 1 C) [20]. In the Fe-S cluster-bound form, containing a [4Fe-4S] cluster, IRP1 is unable to recognise IRE. Instead, it acts as cytosolic aconitase, catalysing isomerisation of citrate to isocitrate [21]. In the apo form, it binds to IRE. Reactive oxygen or nitrogen species, such as NO or H_2_O_2_, degrade the cluster in IRP1, converting it into the apo form. The apo-IRP1 binds to the IRE at the 5´ end of ferritin mRNA to block translation, while at the same time binds to the IRE at the 3´ end of transferrin receptor (TfR) mRNA to stabilise it and increase its translation [22].

In this perspective article, we focus on the emerging function of the [2Fe-2S] cluster in the mitochondrial outer membrane protein mitoNEET as a sensor of molecular oxygen (Fig. 1 C) [23]. This action of the [2Fe-2S] cluster in mitoNEET to sense O_2 _without being degraded is opposed to one of the Fe-S clusters’ roles as a biological fuse, which involves their degradation via reaction with NO, ROS, or O_2_ [1]. We briefly introduce hypoxia and discuss the long-standing paradox in the field regarding how mitochondria sense hypoxia. We discuss emerging evidence linking mitoNEET to mitochondrial function. Finally, we summarise our recent findings suggesting a possible role of the mitoNEET [2Fe-2S] cluster in sensing O_2_level and argue that this function of the cluster provides a mechanistic explanation for the observed mitoNEET role in regulating mitochondrial function and dynamics. These findings and model will have implications for various physiological and pathophysiological conditions, including immune cell function and cancer.

## Hypoxia and mitochondrial function

Hypoxia is defined as low levels of molecular oxygen in a tissue, insufficient for oxidative phosphorylation. It is a common factor in many inflammatory conditions, including infection and cancer [24, 25]. In the cytoplasm, the hypoxia-inducible factors (HIFs) are vital for sensing O_2_ levels and modulating cell response. These proteins are transcriptional regulators that sense cellular O_2_ availability through O_2_-dependent hydroxylation [26]. HIF is a heterodimeric transcription factor composed of two bHLH-PAS proteins: an oxygen-sensitive α subunit (such as HIF1α) and a stable β subunit (such as HIF1β, also known as ARNT1) that binds to hypoxia response elements (HREs) in DNA [26–28]. Under normoxia or normal O_2_ levels, prolyl hydroxylase domain (PHD) enzymes hydroxylate HIF-α subunits, promoting their degradation via the von Hippel-Lindau (VHL) ubiquitin ligase complex [26–28]. As a result, HIF activity remains low when oxygen is sufficient. Hypoxia or oxidative stress inhibits PHDs, which require O_2_, Fe^2+^, and 2-oxoglutarate as cofactors, thereby preventing VHL binding and stabilising HIF-α [26–28]. Then, stabilised HIF-α translocates into the nucleus, dimerises with HIF-β, and binds to HREs in the promoters of target genes. This stabilisation induces expression of genes related to glycolysis, angiogenesis, and inflammation.

**Fig. 2:**
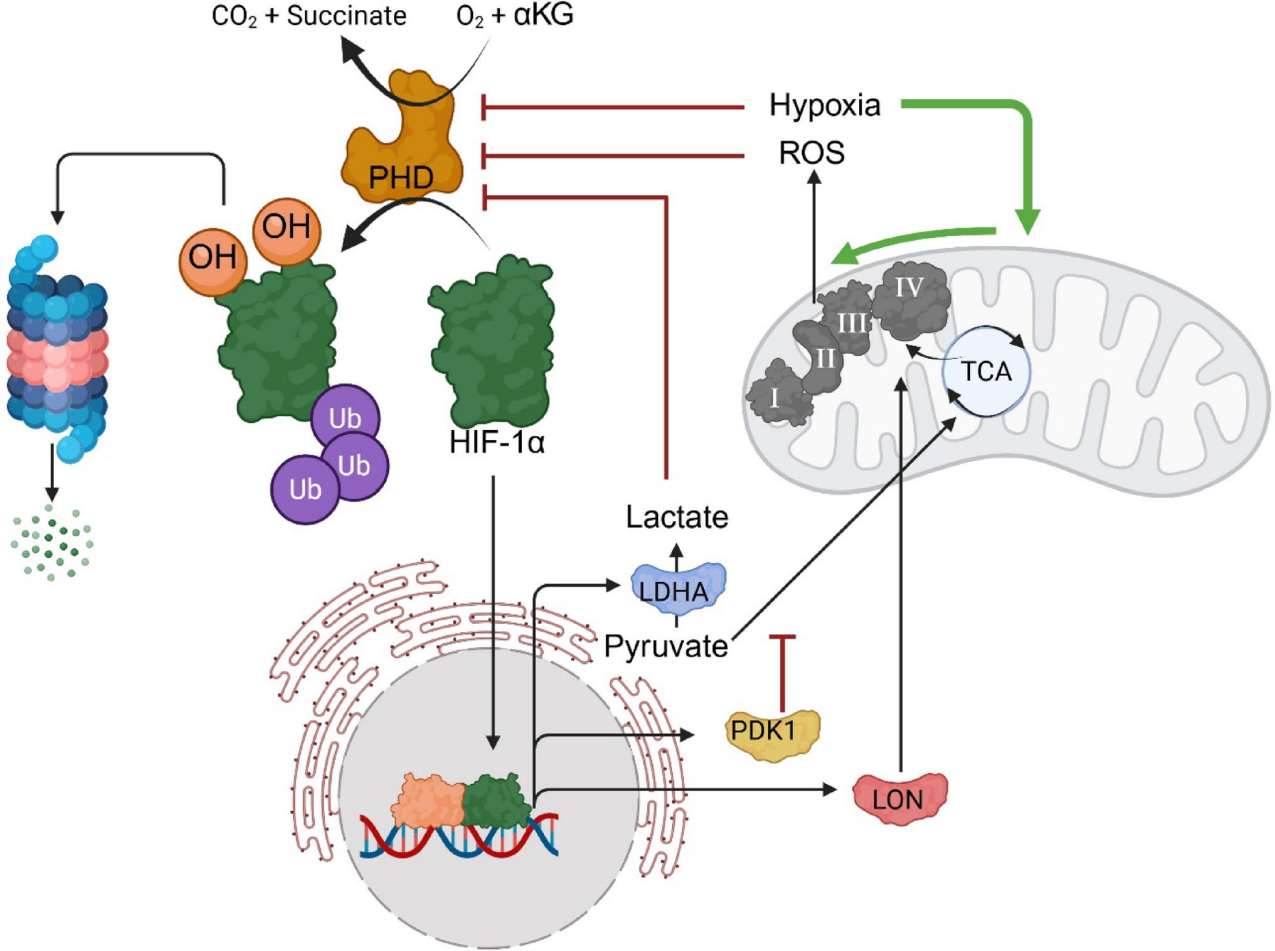
**The impact of hypoxia on mitochondria is multifaceted. **Hypoxia will inhibit the activity of PHD, therefore stabilising HIF-1α, which can activate transcription of multiple cellular proteins, including lactate dehydrogenase (LDHA), phosphoinositide-dependent protein kinase-1 (PDK1), and Lon protease homolog (LON). It is unclear how mitochondria sense hypoxia. The current evidence favours a mechanism by which hypoxia is directly sensed by mitochondria (green arrow). This step leads to biochemical reactions and a reduction in mitochondrial respiration, thus favouring an increase in ROS formation as O_2_ levels decrease

While sensing O_2_ levels in the cytoplasm via HIF is well established, it remains poorly understood how organelles such as mitochondria, whose function is fundamental for immune cells’ activity and plasticity [29, 30], sense O_2_ levels. The link between the HIF pathway and mitochondria is multifaceted (Fig.2) [31]. It is likely regulated by the interplay between O_2_-mediated control of reactive oxygen species (ROS) production and NO synthesis [32]. It is unclear whether ROS production is a consequence of mitochondria sensing hypoxia or a mechanism that signals low O_2_ levels to mitochondria. Available data favour the former. Loss of mitochondrial respiration is generally believed to trigger ROS production [31, 32]. This introduces a paradox: reduced O_2_ concentration and enhanced ROS in hypoxic cells [32]. A solution to this paradox is that reduced O_2_ is initially sensed by mitochondria, triggering biochemical reactions that lead to ROS formation. On the other hand, when various cells experience hypoxic conditions, nitric oxide synthases (NOSs) are activated, therefore increasing the cellular level of NO [33–35]. It is known that NO is a potent competitive inhibitor of O_2_ for binding to cytochrome c oxidase (COX). This inhibition, on the one hand, may help redistribute O_2_ and thus increase PHD activity, which inactivates HIF, and, on the other hand, may increase ROS formation due to decreased COX activity [36]. Consequently, it appears that mitochondrial ROS formation is likely the result of initial sensing of hypoxic conditions. This increase in ROS formation subsequently may further exacerbate oxidative stress by inhibiting the activity of PHD, thereby stabilising HIF-α [31, 37].

## MitoNEET and mitochondrial function

**Fig. 3:**
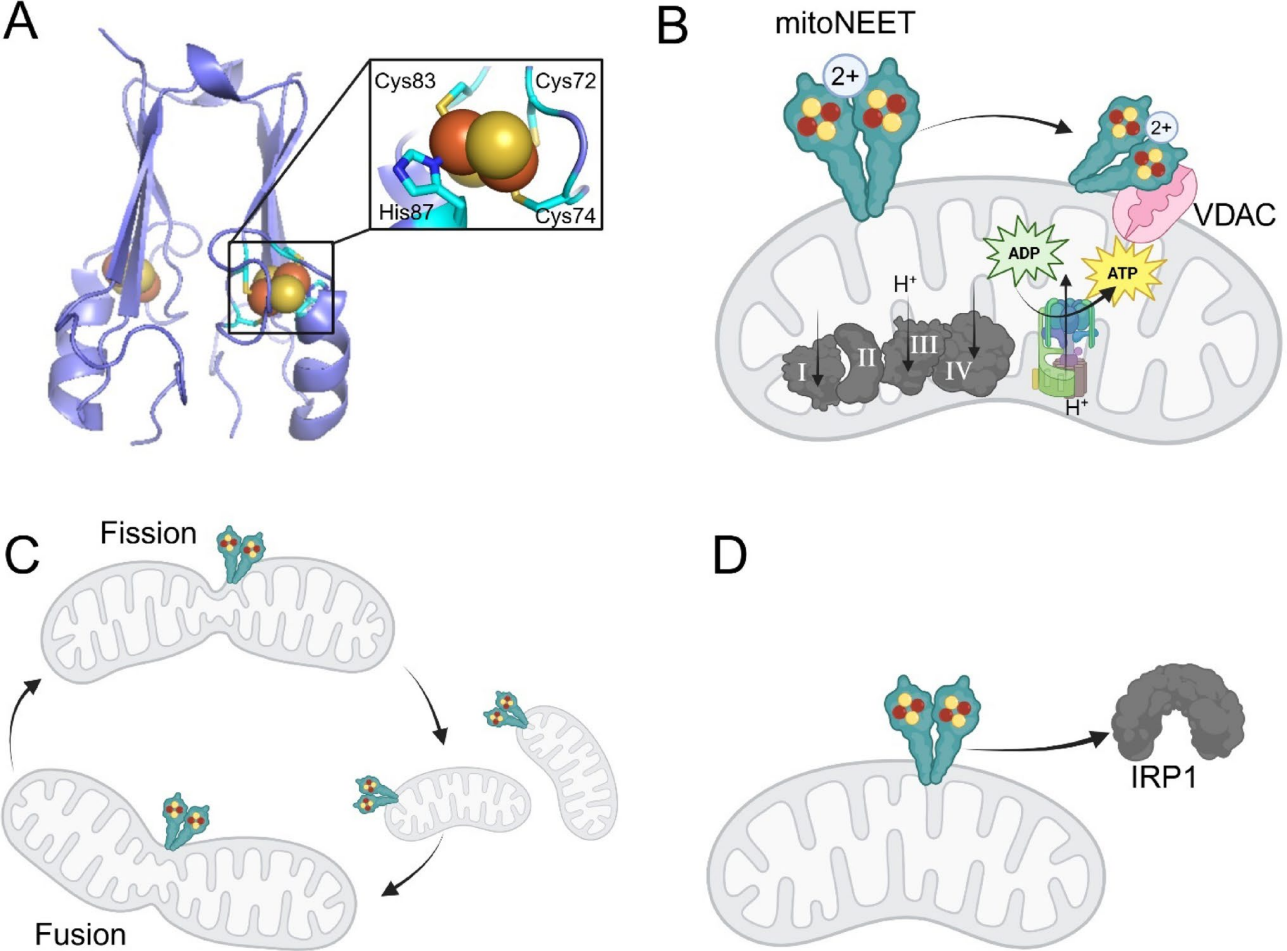
**The mitochondrial outer membrane Fe-S protein mitoNEET plays a key role in mitochondrial bioelectrochemistry**. (**A**) The X-ray crystal structure of mitoNEET (PDB code: 2QH7) shows its [2Fe-2S] cluster. Past findings suggest that mitoNEET (**B**) can interact with VDAC and block its voltage-gating function, (**C**) is involved in mitochondrial fusion and fission, and **(D**) participates in the repair of Fe-S clusters of cytosolic proteins such as IRP1

A possible protein candidate linking sensing of gases such as O_2_ and NO to mitochondrial function is mitoNEET. The NEET (Asn-Glu-Glu-Thr) protein family constitutes a structurally and functionally distinctive class of [2Fe-2S] cluster proteins. Members of this family are defined by a highly conserved CDGSH motif that is involved in coordinating a [2Fe-2S] cluster (Fig.3 A). The coordination environment of this cluster includes three cysteine amino acids and one histidine [38, 39]. Three NEET proteins have been identified: CISD1 (mitoNEET), CISD2 or Wolfram syndrome 2 (NAF-1), and CISD3 (MiNT or Miner2**)** [38–41]. These proteins are different in subcellular localisation. MitoNEET resides at the outer mitochondrial membrane [42], NAF-1 at the endoplasmic reticulum (ER) and ER-mitochondria contact sites [43, 44], and Miner2 within the mitochondrial matrix [45, 46]. While mitoNEET and NAF-1 (CISD2) are homodimers with each monomer containing a [2Fe-2S] cluster, Miner2 (CISD3) is functional as a monomer [38, 45]. The [2Fe-2S] cluster in these proteins reacts with NO and O_2_ [45, 47, 48]. However, how the cluster reacts with these gasotransmitter signalling molecules varies across the NEET family of proteins. For example, the O_2_-resistance of the [2Fe-2S] cluster as a function of pH is not the same. At neutral pH, mitoNEET (CISD1) appears to have the highest O_2_ resistance [45]. However, the molecular mechanism underlying these differences is unclear. The cluster in all proteins has the same coordination environment, three cysteines and a histidine ligand. Therefore, the variation in their reaction with NO and O_2_ is likely related to how these gasotransmitters access the cluster. The cluster is redox-active, but the biological electron donor to the protein is not well-defined. In the case of mitoNEET, it has been shown that the anamorsin/Ndor1 complex can transiently bind to mitoNEET and reduce its cluster [49], and that small molecules such as biological thiols can also reduce the cluster [50].

Accumulating evidence links mitoNEET expression with mitochondrial function and homeostasis. Altering the expression of mitoNEET in adipose tissue changes the ability of these cells to take up and store lipids. When mitoNEET is overexpressed, lipid uptake and storage increase without affecting insulin sensitivity [51]. This role of mitoNEET in regulating fat metabolism is proposed to occur through a mechanism involving mitoNEET expression, which, conversely, regulates iron transport into the matrix [51]. This proposal is based on the transgene induction of mitoNEET expression in adipose tissue. Higher mitoNEET expression was associated with higher β-oxidation rates, which in turn were associated with lower mitochondrial iron content. Therefore, it was concluded that mitoNEET regulates iron transport into the mitochondrial matrix. This model is debatable, as overexpression of mitoNEET via a transgene will increase the cellular demand for [2Fe-2S] cluster synthesis to support mitoNEET expression. This demand would reduce mitochondrial iron content indirectly and affect β-oxidation rates. A more detailed mechanistic study of the mitoNEET Fe-S cluster’s role in mitochondrial function linked the redox state of the cluster to its function in regulating mitochondrial iron content and metabolism through interaction with outer-mitochondrial membrane protein voltage-dependent anion channel 1 (VDAC1) [52]. While reduced mitoNEET was unable to interact with VDAC1, the oxidized mitoNEET interacted with VDAC1 with an nM affinity [52]. This mitoNEET function, acting as a cap on VDAC1, would block metabolic crosstalk between mitochondria and the cytosol, thereby affecting efficient bioenergetics and ATP production (Fig. 3B). Finally, emerging evidence links mitoNEET expression and function to the formation of intermitochondrial junctions (fusion) [53], mitochondrial fission [54] (Fig. 3 C), and the repair of the Fe-S cluster of cytosolic client proteins, including IRP1 (Fig. 3D) [55]. The exact mechanism underlying these roles is unclear. A potential function of mitoNEET in mitochondrial fusion/fission will likely regulate mitochondrial homeostasis and its activity under different conditions [56]. Hence, interference with these functions of mitoNEET will disturb respiration and can lead to loss of mitochondrial membrane potential (*ΔΨ*_*m*_).

## Insight into the mechanism of O_2_ sensing by mitoNEET

**Fig. 4:**
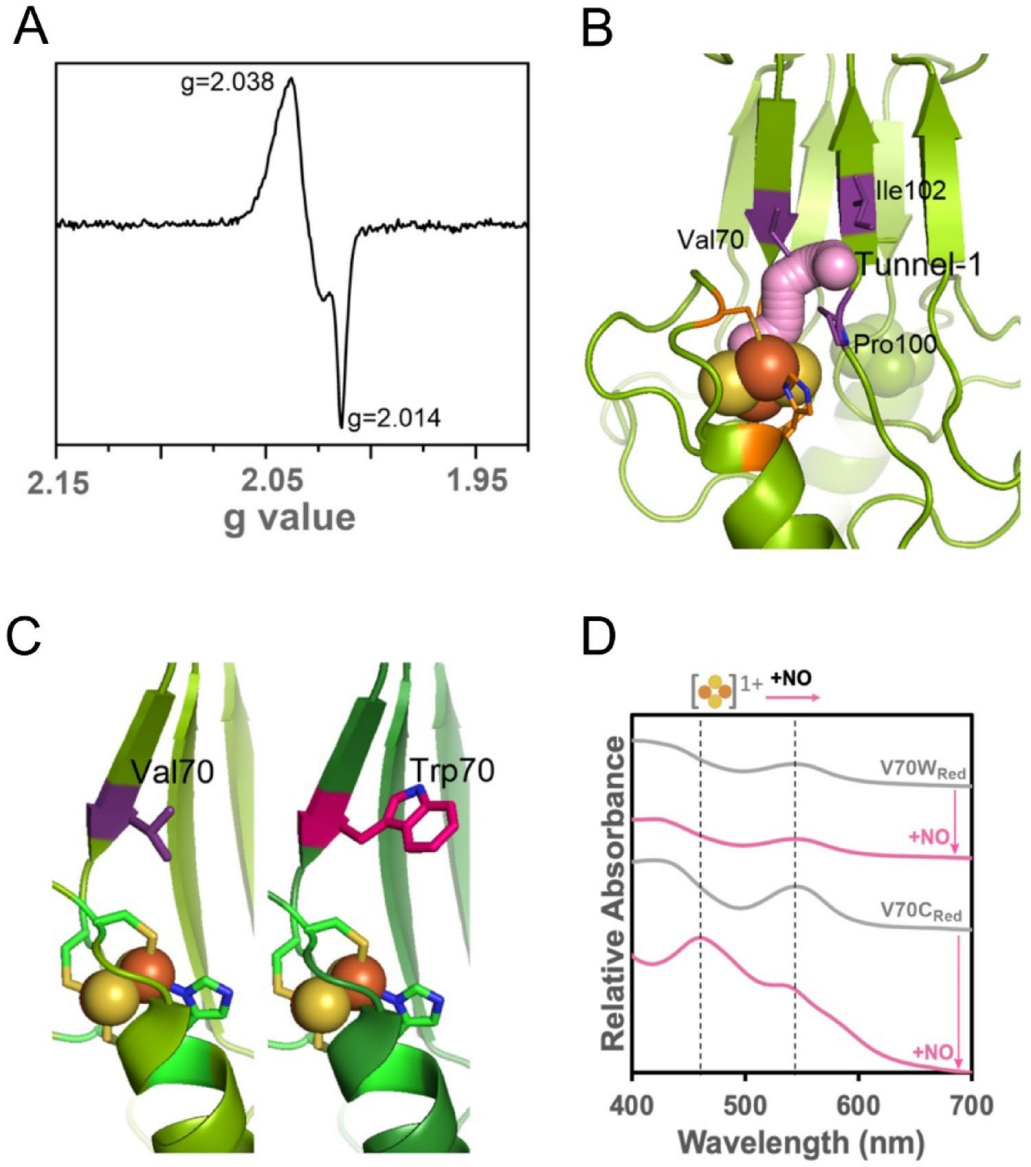
**Identification of a potential gas tunnel regulating mitoNEET [2Fe-2S] cluster reaction with gasotransmitters.** (**A**) The formation of an EPR-active iron-nitrosyl species after exposure of mitoNEET to NO-releasing agent. (**B**) Computational studies predict a short gas tunnel exposing the cluster. The figure highlights the key residues forming the entry to the gas tunnel. (**C**) Mutation of Val70 to tryptophan (**D**) limits NO access and oxidation of reduced [2Fe-2S]^1+^ cluster by NO, but mutation of Val to cysteine does not limit NO access to the cluster and its subsequent oxidation. The data are taken from [23] with permission

Despite extensive prior studies linking the mitoNEET oxidation state to mitochondrial activity, e.g., by gating VDAC function, and its cluster sensitivity to gasotransmitters such as NO and O_2_, it remained unclear how exactly the mitoNEET [2Fe-2S] cluster reacts with gasotransmitters, and what is the underlying mechanism linking reaction with gasotransmitters, mitoNEET oxidation state, and mitochondrial activity. To address these questions, we studied the interplay between the mitoNEET [2Fe-2S] cluster reaction with different gasotransmitters involved in the immune system, namely, NO, O_2_, and H_2_S, and we investigated how these small-molecule gases reach the cluster [23]. We first studied the reaction of NO with the mitoNEET [2Fe-2S] cluster, compared with the [4Fe-4S] cluster of hSAND (RSAD2 or viperin), under anaerobic conditions. We used UV-visible spectrophotometry, liquid chromatography-mass spectrometry (LC-MS), and continuous-wave electron paramagnetic resonance (EPR) spectroscopy. This study provides information on how the protein environment is likely to affect the Fe-S cluster’s sensitivity to reaction with NO. While the [4Fe-4S] cluster of SAND was highly sensitive to NO exposure, leading to enzyme inactivation, the mitoNEET [2Fe-2S] cluster was not degraded. The chemical reducing agent, sodium dithionite, reduced the NO-exposed and oxidized [2Fe-2S]^2+^ cluster back to the reduced [2Fe-2S]^1+^ cluster. Additionally, NO exposure increased the absorbance intensity of the [2Fe-2S]^2+^ cluster, suggesting a potential direct interaction between NO and the cluster. To further test the possibility that NO directly interacts with the [2Fe-2S]^2+^ cluster of mitoNEET, we applied EPR spectroscopy. We detected a paramagnetic species with an EPR spectrum identical to that reported for iron-nitrosyl complexes (Fig. 4 A) [57], supporting a direct interaction between NO and the [2Fe-2S] cluster.

Although the binding of NO to the [2Fe-2S] cluster of NEET proteins has been described [47, 58], the mechanism by which NO reaches the cluster remained unknown. To address this question, we applied advanced computational biochemistry, including AI-based structural modelling and all-atom molecular dynamics simulations combined with mutagenesis studies [23]. These analyses revealed a short tunnel connecting the protein surface to the [2Fe-2S] cluster (Fig. 4B), providing a potential diffusion path for NO to reach the cluster. This prediction was further supported by molecular dynamics simulations, which showed that NO could diffuse directly towards the cluster, whereas H_2_O_2_, which is not a gas, did not. Guided by this computational prediction, we designed mitoNEET variants in which Val70, an amino acid residue forming the entry of the gas tunnel to the mitoNEET cluster, was substituted by Trp70 (V70W) (Fig. 4 C) or Cys70 (V70C). The wild-type and mutant proteins were purified and characterised. UV-visible absorbance spectroscopy was employed to monitor changes in the redox state of the [2Fe-2S] cluster upon exposure to NO and H_2_S. Unlike the wild-type mitoNEET, where NO could oxidise the reduced [2Fe-2S]¹⁺ cluster, this reaction did not occur in the V70W variant (Fig. 4D). In contrast, NO oxidised the [2Fe-2S]¹⁺ cluster in the V70C variant (Fig. 4D). These observations suggest that replacing the tunnel residue Val70 with the larger Trp70 effectively blocks NO from reaching the cluster, supporting the idea that this tunnel acts as a functional entry route for gaseous ligands.

**Fig. 5:**
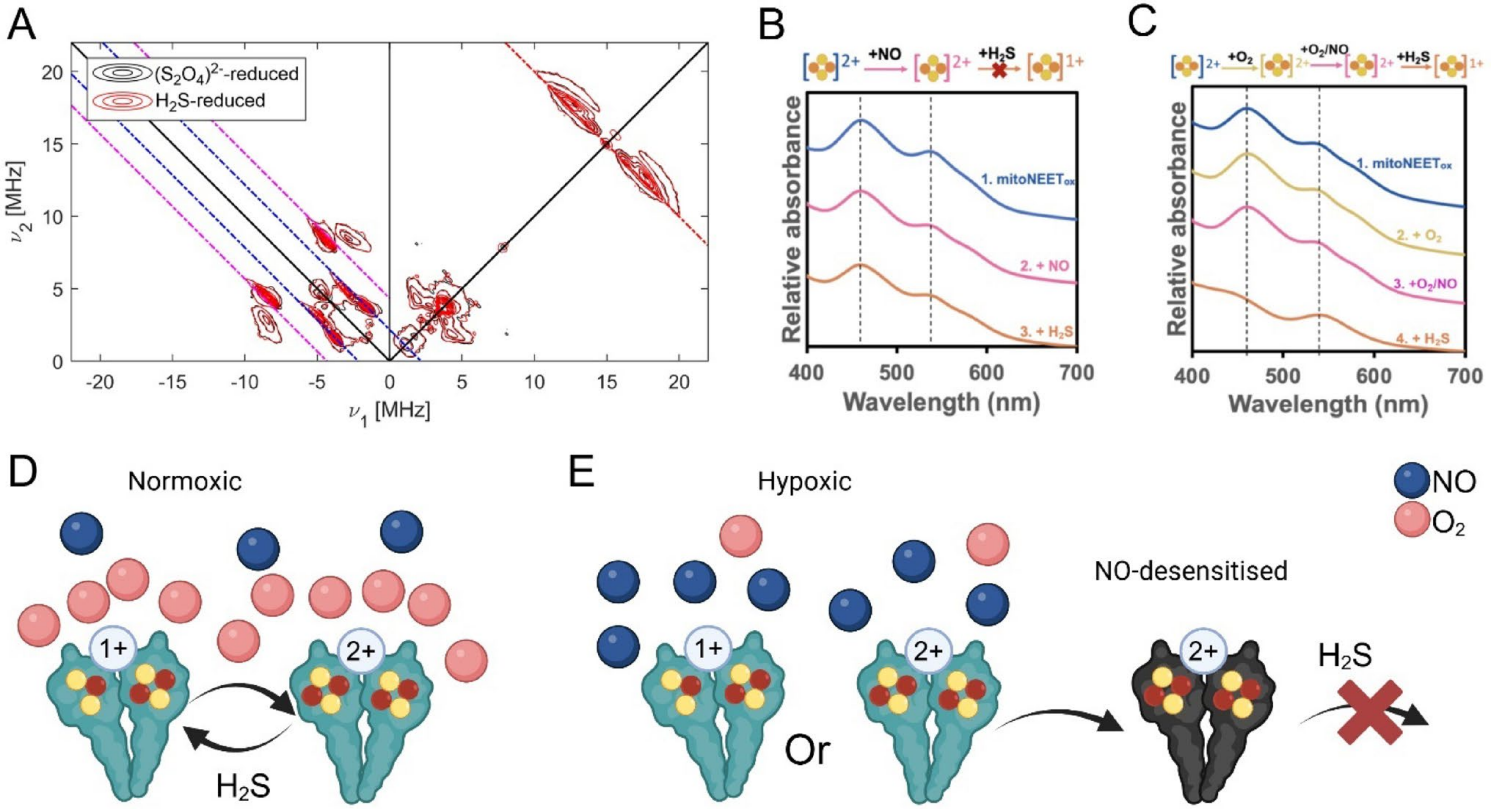
O_2_ protects the mitoNEET [2Fe-2S] cluster from NO reaction and its desensitisation to H_2_S reduction. (**A**) The HYSCORE spectrum of the H_2_S - and dithionite-reduced [2Fe-2S]^1+^ cluster confirms that the reduction of the cluster by H_2_S does not affect its coordination environment. (**B**) H_2_S cannot reduce the NO-exposed mitoNEET [2Fe-2S]^2+^ cluster. (**C**) When O_2_ is present, NO cannot react with the cluster, and consequently, the oxidized [2Fe-2S] cluster is reduced by H_2_S. (D-E) The proposed model for mitoNEET roles in sensing hypoxia. (**D**) Under normoxic conditions, the cluster is protected by O_2_, and there is a balance between the reduced and oxidized forms of mitoNEET. (E) Under hypoxic conditions, however, the cluster reacts with NO and is desensitised to reduction by H_2_S and potentially other biological reducing agents. Consequently, the levels of oxidized and desensitized mitoNEET increase, which can serve as a signal for mitochondrial function. (A-C) Data are taken from [23] with permission

Having established a NO-access tunnel to the [2Fe-2S] cluster of mitoNEET, we sought to determine whether the gasotransmitter hydrogen sulfide (H_2_S) [23], which plays a role in mitochondrial metabolism and redox signalling [59–61], could access the cluster and reduce it. To test this, we exposed purified mitoNEET to H_2_S under anaerobic conditions. Using UV–visible absorbance spectroscopy, along with continuous-wave electron paramagnetic resonance spectroscopy (CW-EPR) and Hyperfine sublevel correlation (HYSCORE) spectroscopy, we observed that H₂S reduced the oxidised [2Fe-2S] cluster without altering its coordination environment (Fig. 5A). These spectroscopic data confirmed that H₂S acts as a reducing agent while leaving the cluster intact. In contrast to this reaction, H_2_S was unable to reduce the [4Fe-4S] cluster in the radical-SAM enzyme human SAND. Therefore, H_2_S’s ability to reduce the mitoNEET cluster appears to be specific. To further explore the interplay between NO and H_2_S, we tested whether NO could oxidize the H_2_S-reduced cluster or H_2_S could reduce the NO-exposed cluster [23]. Stoichiometric amount of an NO-releasing agent, relative to mitoNEET concentration, was added. After consumption of the NO-releasing agent and after any remaining NO gas fully escaped from the reaction during overnight incubation in the glovebox, the solution was exposed to the H_2_S-releasing agent. The results showed that H_2_S could not reduce the NO-exposed cluster. When samples were prepared by exposing the mitoNEET first to H_2_S and subsequently to NO, the H_2_S-reduced mitoNEET [2Fe-2S]^1+^ cluster was oxidized by NO (Fig. 5B). The results together indicate that NO effectively desensitises the cluster to reduction by biological reducing agents like H_2_S.

We next examined how O_2_ modulates the interaction between NO and the cluster. MD simulations revealed that the O_2_ survival probability (defined as the fraction of ligands that remain bound to the protein over time) in the gas tunnel is higher than that of NO. This finding suggested that O_2_ could potentially block NO access to the cluster. To experimentally validate this prediction, we leveraged our finding that H_2_S cannot reduce the NO-exposed and desensitised [2Fe-2S] cluster. Purified mitoNEET was first exposed to O_2_, and subsequently, NO-releasing agents were added to the mixture. We confirmed that adding the NO-releasing agent under an oxygenic environment still provides sufficient NO to react with the cluster. Subsequently, the sample was taken to the glovebox and, after removal of NO, was treated with H_2_S. Under these conditions, H_2_S reduced the cluster, suggesting that O_2_ prevented the NO reaction with the cluster, as computational studies predicted (Fig. 5C). We observed that pioglitazone, a known type-2 diabetic drug that targets mitoNEET [62], protected the mitoNEET [2Fe-2S] cluster from NO-induced desensitization. Molecular docking studies showed that pioglitazone binds to the amino acid residues forming the entry to the gas tunnel and thus, can block NO access to the cluster, very similar to O_2_. Consistently, in the presence of pioglitazone, NO exposure did not lead to desensitization of the cluster, as H_2_S was able to reduce the cluster.

Taken together, our biochemical analysis revealed new insights into the role of the mitoNEET [2Fe-2S] cluster in sensing gasotransmitters (NO, O_2_, and H_2_S) and into a potential role of O_2_ in protecting the cluster. Based on these findings, we postulate a new model for how mitochondria might sense O_2_. Under normoxic conditions, O_2_ molecules prevent NO from accessing the [2Fe-2S] cluster, thereby maintaining mitoNEET in a redox-active state and allowing it to cycle between reduced and oxidized states (Fig. 5D). Under hypoxic conditions, as oxygen levels fall, O_2_ occupancy diminishes, allowing NO to penetrate the tunnel and react with the cluster. NO reaction with the cluster leads to the formation of iron-nitrosyl species, thereby affecting the cluster’s redox potential and desensitising it to reduction by the gasotransmitter H_2_S or potentially other biological reducing partners (Fig. 5E). Consequently, the concentration of desensitised and oxidised mitoNEET at the mitochondrial outer membrane increases. This increase serves as a signal of reduced cytoplasmic molecular oxygen levels.

## Concluding remarks

**Fig. 6:**
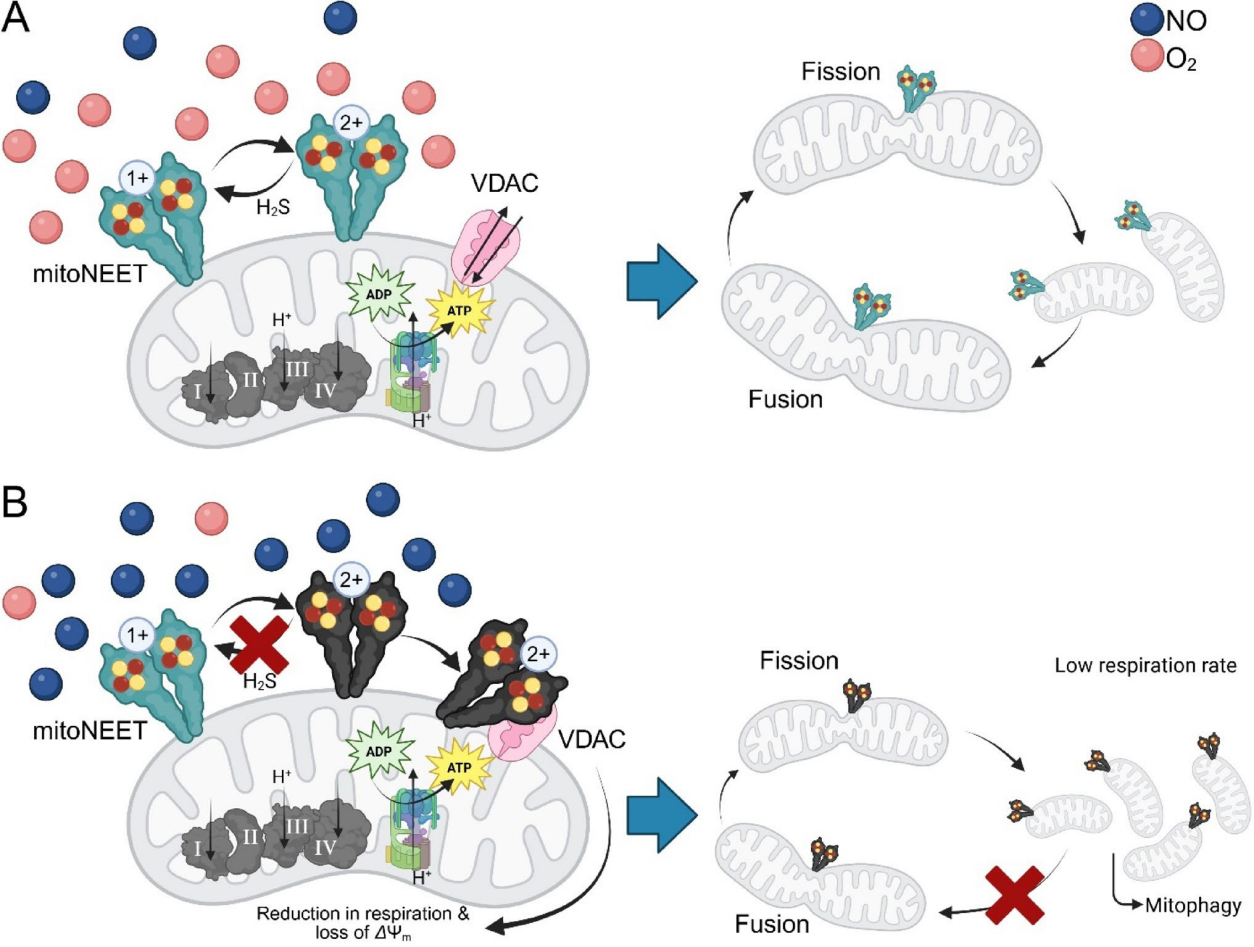
**The proposed model shows how the oxidized mitoNEET might act as a hypoxia signal for mitochondria.** (**A)** Under normoxic conditions, the mitochondrial membrane potential (ΔΨ_m_) is normal, and consequently, mitochondrial fusion and fission are regulated to adapt to the cells’ respiratory demand. (**B**) Under hypoxic conditions, the level of oxidized and desensitised mitoNEET (black) increases. It blocks the voltage-gating activity of VDAC1, leading to a loss of membrane potential (ΔΨ_m_). Consequently, the fusion is disrupted. Some of the damaged mitochondria may undergo mitophagy or other biological processes [68].

Our proposed role of the mitoNEET [2Fe-2S] cluster as a sensor of the cytoplasmic level of molecular oxygen links the mitoNEET oxidation state to its downstream impact on mitochondrial activity and homeostasis, thereby providing a mechanistic explanation for how mitochondria might sense hypoxia (Fig. 6). Under hypoxic conditions, O_2_ levels decrease, and NO synthesis increases. Consequently, the level of NO-oxidised and desensitized mitoNEET increases. This oxidized form cannot be reduced readily by H_2_S or other reducing partners, and thus it accumulates on the mitochondrial membrane. The accumulation of oxidized mitoNEET can be linked to different biological activities reported for this protein. Firstly, the oxidized mitoNEET will bind to the voltage-dependent anion channel (VDAC) and inhibits its redox gating function. Binding to VDAC inhibits ADP/ATP exchange, reducing mitochondrial respiration and membrane potential (ΔΨ_m_), which activates PINK1 [63]. Activated PINK1 phosphorylates PARKIN, which ubiquitinates target proteins at the outer membrane, thereby promoting mitophagy to eliminate dysfunctional mitochondria [64–66]. Secondly, the accumulation of oxidized and NO-desensitised mitoNEET on the mitochondrial outer membrane might interfere with the proposed role of mitoNEET in the formation of intermitochondrial junctions [53]. The formation of these junctions is fundamental for mitochondrial fusion [67], a vital process for cell life and death, and linked to age-associated neurodegenerative diseases [68]. Disturbance of the mitochondrial fusion may act as a signal for damaged mitochondria and mitophagy [68]. The sensing of low O_2_ by mitoNEET and its impact on mitochondrial ΔΨ_m_ and fusion can increase ROS formation. Ligands like pioglitazone can protect mitoNEET against NO-desensitization and oxidative damage, thereby restoring mitochondrial function and homeostasis, and demonstrating the therapeutic potential of targeting this O_2_-sensing mechanism.

Future studies should investigate at the cellular level the proposed role of mitoNEET in sensing O_2_ levels and the link between this function and downstream biological activities. These studies will help fully understand how the mitoNEET [2Fe-2S] chemistry, oxidation state, and its reaction with gasotransmitters are linked to mitoNEET function in regulating mitochondrial activity and homeostasis. The outcomes will help develop new therapeutics targeting mitochondrial dysfunction associated with neurodegeneration and the aging immune system.

## Data Availability

All data analyzed during this study are included in the article.
